# Naturalised *Vitis* Rootstocks in Europe and Consequences to Native Wild Grapevine

**DOI:** 10.1371/journal.pone.0000521

**Published:** 2007-06-13

**Authors:** Nils Arrigo, Claire Arnold

**Affiliations:** 1 Laboratory of Evolutionary Botany, University of Neuchâtel, Neuchâtel, Switzerland; 2 National Centre of Competence in Research (NCCR) Plant Survival, University of Neuchâtel, Neuchâtel, Switzerland; Purdue University, United States of America

## Abstract

The genus *Vitis* is represented by several coexisting species in Europe. Our study focuses on naturalised rootstocks that originate in viticulture. The consequences of their presence to the landscape and to native European species (*Vitis vinifera* ssp. *silvestris*) are evaluated. This study compares ecological traits (seven qualitative and quantitative descriptors) and the genetic diversity (10 SSR markers) of populations of naturalised rootstocks and native wild grapevines. 18 large naturalised rootstock populations were studied in the Rhône watershed. Wild European grapevines are present in four main habitats (screes, alluvial forests, hedges, and streamside hedges). In contrast, naturalised rootstock populations are mainly located in alluvial forests, but they clearly take advantage of alluvial system dynamics and connectivity at the landscape level. These latter populations appear to reproduce sexually, and show a higher genetic diversity than *Vitis vinifera* ssp. *silvestris*. The regrouping of naturalised rootstocks in interconnected populations tends to create active hybrid swarms of rootstocks. The rootstocks show characters of invasive plants. The spread of naturalised rootstocks in the environment, the acceleration of the decline of the European wild grapevine, and the propagation of genes of viticultural interest in natural populations are potential consequences that should be kept in mind when undertaking appropriate management measures.

## Introduction

The genus *Vitis* is represented by several coexisting species in Europe. *Vitis vinifera* L. ssp. *silvestris* (Gmelin) Hegi is the only extant wild European taxon.

Many spontaneous forms of grapevine cultivars are also naturalised in Europe. They belong to *V. vinifera* L. ssp. *vinifera*, introduced for at least a thousand years when domesticated forms of grapevine were spread throughout Europe [1]. Several American and Asian *Vitis* species have been introduced during the last century as rootstock.

In this paper we will only focus on the naturalised rootstocks and the native European wild grapevines present in natural environment.

Rootstocks were introduced to Europe after the phylloxera invasion, a pest which rapidly spread through vineyards, destroying large areas of sensitive cultivars. Grafting European varieties on pathogen-resistant rootstock is now a normal procedure and many varieties of rootstock have been developed by breeders. The more common American species used for this purpose are: *Vitis riparia* Michaux, *Vitis rupestris* Scheele, *Vitis rotundifolia* Michaux, *Vitis berlandieri* Planchon and *Vitis labrusca* L.. Other species from Europe (*V. vinifera* L.) and Asia (*Vitis amurensis* Ruprecht) are also used. Several traits have been selected by breeders, such as resistances to phylloxera (*V. riparia*, *V. rupestris* and *V. berlandieri*), nematodes, drought (hybrids *berlandieri-rupestris*), limestone (*V. vinifera*), salt and frost (*V. amurensis*).

American species are known to easily interbreed and barriers to hybridisation are mainly phenological [2], [3]. Location of glacial refugia, low contrasted landscape, human influences such as cattle farming, fire and forest management have induced sympatry which favours hybridisation of different *Vitis* species in the central United States [3], complicating the morphological identification of species.

*Vitis* species are known to play an important role in plant communities in the United States. Two species are reported to produce numerous long-living seeds (*V. aestivalis* and *V. rotundifolia*, [4]), which are able to germinate even five years after burial in the forest floor. Moreover, their quick and thick growth is able to effectively change plant communities [5]. These species are most abundant in moderate to highly disturbed locations. Early stages in forest development seem to be especially suitable, but individuals covering mature trees can also occur. *V. rotundifolia* is reported to be widespread and not associated with any specific ecosystem [6]. However, three of the main *Vitis* species used in rootstock breeding programmes are restricted to streambeds and gullies (*V. riparia, V. rupestris* and *V. berlanieri* [5]). Their presence in floodplains allows American species to benefit from landscape connectivity created by streams and rivers [7], [8]. In this way, they also partly overlap the ecological niche of European *V. vinifera* ssp. *silvestris* [9].

*V. vinifera* ssp. *silvestris* has been used by humans since the Early Neolithic, as shown by the amounts of pips recorded in prehistorical sites, in caves and along lakes [2]. Cultivation of wine or table grapes was developed four to six thousand years ago in Transcaucasia and rapidly spread out in the Near East and later in southern Europe, following trade routes [1], [2]. Several authors have suggested that native wild grapevines have been involved in local domestication events during the spread of viticulture in Europe [1], [2], [10]–[12]. This wild taxon is still considered as a gene pool for viticulture [13].

Because of the recent loss of suitable habitats due to direct and indirect human impact, *V. vinifera* ssp. *silvestris* is now endangered throughout its range. Its distribution across Europe has been drastically reduced [9] and it is therefore legally protected in some European countries. The two main known factors threatening wild grapevine populations are diseases issued from viticulture and eradication of wild grapevines through forest and river management [14], [15]. Moreover, the human-driven deepening of the water table allows grapevine pests and diseases to enter within the floodplain forests, and other woody climbers to become more competitive [9]. As a consequence, populations are generally small and dispersed, with about five individuals per population in average (pers. obs.).

Our study combines both ecological and genetic approaches to better understand the current status of *Vitis* populations in wild settings in Europe. This study aims to outline occurrences of naturalised rootstock away from viticulture areas, to define the overlap of ecological niches between native and naturalised *Vitis* groups, to give insights about the escape processes and creation of networks between naturalised populations, and to evaluate the spread potential of naturalised populations, especially by considering viticulture as regular source of genotypes escaping.

## Results

### Ecology

**Figure 1:**
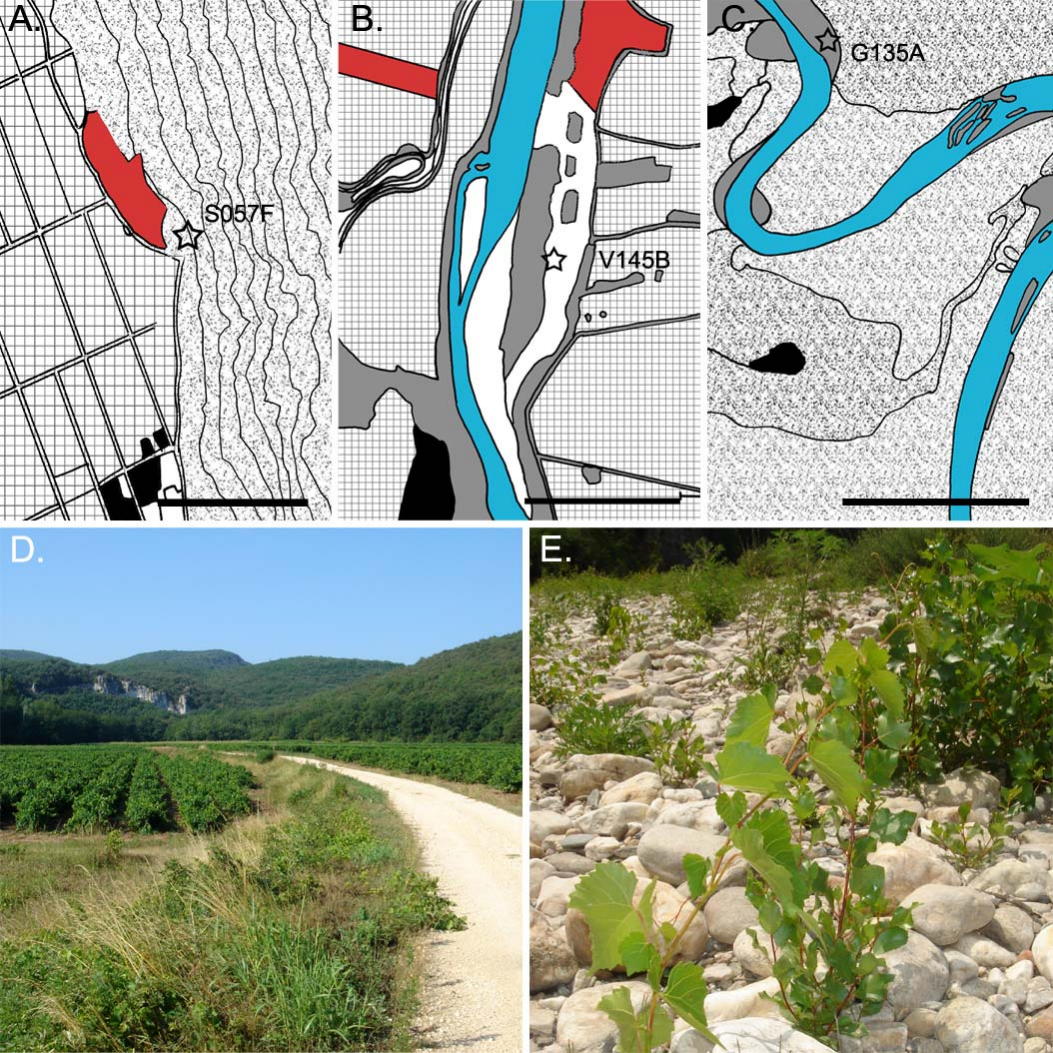
Maps of naturalised rootstock populations (based on Map S1). The stars represent the naturalised rootstock populations. Landscape structures are indicated (red–vineyards, patches of points–forests, grey–floodplain vegetation, squares–crop fields and black–habitations). The black scale bar is 500 meters long. A. Anthropogenic escaped population located in Switzerland. The escaped individuals are less than 50 meters away from the vineyard. B. Intermediate site between anthropogenic and natural escaped population. Naturalised rootstock individuals are 500 meters away from the vineyard, and begin to occupy areas subject to flooding. C. Natural escaped population. The naturalised rootstock individuals are no longer linked to vineyards and have colonised the river bed in a stream curve. D. Escaping individuals along a road nearing vineyards. E. Seedlings of naturalised rootstocks growing on the riverbed of a stream in a natural escaped population.

A total of 24 populations of naturalised rootstocks were identified at various distances from vineyards (Figure 1 A. to C. and Map S1). In these populations, the number of adult individuals ranged from one to over hundred. Additionally seedlings and young plants were regularly observed (Figure 1 D. and E.).

**Figure 2:**
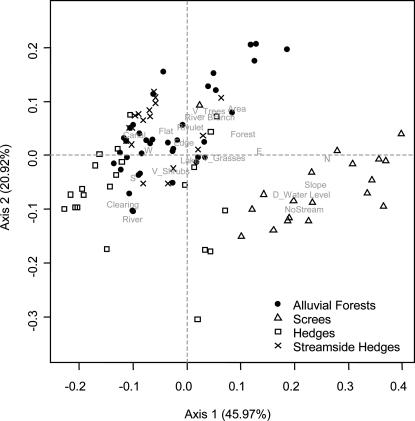
Scatterplot of the PCoA analysis of the ecological dataset (Gower similarity index). The two first axes display 45.97% and 20.92% of the total variance. Individuals (in black) are labelled according to their habitat (Alluvial Forest, Scree, Hedge, and Streamside Hedge). A total of 20 variables (in grey) are used: slope (%, quantitative), exposition (NSEW or flat, binomial categories), area covered by the population (m^2^, quantitative), vegetation type (forest, forest edge or clearing, binomial categories), vertical structure of the grapevine on tree, shrub or grass strata (V_Trees, V_Shrubs and V_Grasses, semi-quantitative), distance to the water level (m, quantitative), type of stream (river, rivulet, canal, river branch, lake or absence of water nearby, binomial categories).

Populations in screes and alluvial forests were discovered (Figure 2), representing known habitats of the wild grapevines [9], [16]. In addition, two new habitats were discovered and designated as hedges and streamside hedges. These two habitats generally occur in open areas, with a similar vegetation structure containing a single row of trees surrounded by shrubs. They essentially differ from screes and alluvial forests by the geomorphology of the site (low slope and small area covered by the population) and the growth strategy of the grapevines which are positively linked to shrub and grass strata (V_Grass and V_Shrubs). Distance to water level discriminates streamside hedges from hedges. Streamside hedges are commonly found along canals, they differ from alluvial forests by the low alluvial activity of the stream.

*V. vinifera* ssp. *silvestris* is present in all four habitats, with a regular frequency (Table 1). Naturalised rootstocks are abundant in alluvial forests but absent from the screes. One naturalised rootstock population is outlined in hedges and one in streamside hedges (Table 1).

**Table 1:** Occurences of *Vitis vinifera* ssp. *silvestris* versus naturalised rootstock populations in the four different habitats.

	***V. vinif. silvestris***	Rootstocks	
***Occurences (populations)***			**Total**
**Alluvial Forests**	10	16	26
**Screes**	12	0	12
**Hedges**	10	1	11
**Streamside Hedges**	5	1	6
**Total**	37	18	55

The sampling represents 124 individuals collected in 55 populations.

### Genetics

**Figure 3:**
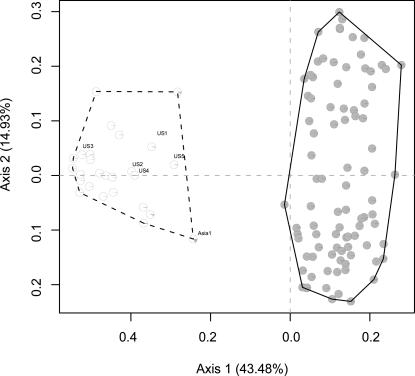
Scatterplot displaying genetic data. Three methods are compared in order to identify individuals: ordination (PCoA calculated on a Jaccard similarity matrix), non-hierarchical clustering (K-means, with two groups assumed) and Bayesian clustering (Structure 2.0, with admixture model). The main axis displays 43.48% and 14.93% of the total variance. K-means groups are represented with polygons surrounding individuals belonging to the rootstock group (dashed line) or *V. vinifera* ssp. *silvestris* group (entire line). Bayesian probabilities are represented with pie charts. The white part of the pie represents the probability to belong to the rootstock group, while the grey part the *V. vinifera* ssp. *silvestris* group. Six common cultivated rootstocks were included in the analysis (Asia1–*V. amurensis*, US1–SO4 cl 5, US2-Rupestris du lot cl 110, US3-Riparia gloire cl 1, US4-Richter 110 cl 7, US5–*V. aestivalis*). Hybrids between both groups are clearly absent. Moreover, none of the 23 naturalized rootstocks collected were a true-to-type clone of the cultivated rootstocks included in the analysis.

Naturalised rootstocks and *V. vinifera ssp. silvestris* individuals clearly belong to two distinct genetic pools. Three methods (PCoA, K-means and Bayesian clustering) were used to investigate the genetic dataset without detecting intermediate individuals (Figure 3).

The genetic profile of each naturalised rootstock was compared to a representative subset of rootstock clones cultivated in the Rhone Valley. No collected individual appeared to be a true-to-type rootstock clone, according to our reference set based on 20 of the most common clones in Switzerland and France. Moreover, 19 different genotypes out of the 23 analysed individuals were discovered.

Rootstocks clearly have a broader genetic diversity, compared to *V. vinifera* ssp. *silvestris* (Table 2). Observed heterozygosities (Ho) are higher for the rootstock (Δ = 0.14), but this result is not significant. The genetic diversity indexes such as allelic richness (Δ = 5.70***) and Shannon's index (Δ = 0.64***) are significantly higher for rootstocks. Variance in allele sizes details a wide variety of alleles for rootstock (VarRepSSR = 22.45, Δ = 14.56*).

**Table 2:** Compared genetic diversity indices of *Vitis vinifera* ssp. *silvestris* versus naturalised rootstock populations.

	***V. vinif. silvestris***	Rootstocks	Δ	p-value
**N.indiv**	101	23	—	—
**Ho**	0.58	0.72	0.14	0.106
**Rs**	6.5	12.2	5.70 ***	0.002
**Shannon Index**	1.35	1.99	0.64 ***	0.003
**VarRepSSR**	7.89	22.45	14.56 *	0.049

N.indiv–Number of individuals included in the analysis, Ho-observed heterozygosity, Rs-Allelic Richness per locus, Shannon Index of diversity, VarRepSSR-variance in allele sizes, Δ is difference between the statistics of both taxa, p-value is calculated with the non-parametric Wilcoxon test. The sampling is exhaustive for populations of *V. vinifera* ssp. *silvestris* and one to four individuals were collected in the escaped rootstock populations.

## Discussion

### Ecology

Screes are generally unlikely to be invaded by alien species [17]. Indeed, naturalised rootstocks have not yet been found in such environments despite populations being located in viticulture regions. This situation is typically observed in the Swiss Alps where viticulture had to exploit hillsides up to an altitude of 1000 meters. Screes are generally spatially isolated, which limits the income of naturalised rootstocks to zoochory. Establishment of new populations then depends on seed reproduction. Soil conditions, colluvial activity, or competition with local adapted species, such as brambles, may explain the absence of seedlings of naturalised rootstocks in screes.

*V. vinifera* ssp. *silvestris* is adapted to screes [18]. Their spatial isolation protects these locations from direct human disturbances. Introduction of pests such as phylloxera is therefore hampered by the ecological features specific to screes. Unfortunately, current wild grapevine populations are usually small and isolated from each other (pers. obs.). Many populations also seem to be senescent and their future mainly relies on vegetative reproduction (pers. obs.). Anthropogenisation of alpine alluvial regions and the systematic eradication of wild grapevine populations by forest management may have played a central role in this isolation. Consequently, the existing connectivities between alluvial forests and screes [19], [20] was drastically reduced if not halted.

Hedges are a new habitat revealed by our study. In many aspects, they can be considered as functional extensions of other known habitats but with a different vegetation structure. These habitats are arranged in corridors in the landscape, promoting dispersal of ornithochorous species [21].

A single naturalised rootstock population was found in hedges. However, its presence shows that some rootstock individuals are able to settle in such habitats. Many naturalised individuals were observed in vineyard borders, but they were not included in the present study as we only focused on habitats distant from viticulture.

Both non-European and native *Vitis* species are largely present in alluvial forests. Vegetative reproduction and abiotic-driven seed dispersal are, in our opinion, implicated in streamside habitats, like alluvial forests or streamside hedges. Streams disperse seeds, pruning wastes, or broken branches to new alluvial locations, promoting settlement of new populations along the alluvial network (pers. obs.). These new sites depend on the geomorphology of the river. Former riverbeds or stream curves are especially suitable for new population establishment [5]. Additionally, floods regularly remove parts of the vegetation and create open areas, covered with sand, that are free of competition. For instance, gravel bars in alluvial zones are known to be suitable sites for alien species [22].

At the convergence of small streams, the main rivers may also act as collectors of naturalised species, and generally shelter naturalised rootstocks proliferations.

Many discovered sites may originate from such dispersal strategies as they were found close to large vegetal deposits accumulated during flooding. Along the original Rhone riverbed, in Lapalud (84), a dense cover of rootstock seedlings was observed on the ancient riverbeds. In that case, the large flood of the winter 2003–2004 may have played a central role in the long-distance abiotic-driven seed dispersal event. Similar occurrences were found along the main tributaries of the Rhone and Durance rivers (Codollet (31), Baix (07) or Villeurbanne (69)). This process has also been documented in European grapevines [9].

Naturalised rootstocks undoubtedly originate in viticulture areas. Their escape process is not yet documented. We suggest two origins: Anthropogenic escaped populations, and natural escaped populations. The two functional types of naturalised populations are linked, and belong to a source-sink metapopulation system. The transition between the anthropogenic and natural escaped populations is recognizable on vegetation maps (figure 1, A. to C. and Map S1).

Anthropogenic escaped populations represent directly contaminated zones and originate from management of viticulture. These populations generally cover important surfaces and constitute active sources of escaping individuals. Vineyard edges and abandoned vineyards belong to this category. These areas and their surroundings are often covered with bushes colonised by rootstocks [23].

Genetics of such populations are expected to vary greatly depending on the considered naturalised rootstock. The official rootstocks in France [24] are dioecious, but both sexes are allowed in vineyards. For instance, Riparia gloire de Montpellier, Rupestris du lot, Teleki 5C and SO4 are common male clones. Anthropogenic escaped populations constituted with these rootstocks are expected to be clonal since only vegetative reproduction can multiply individuals. 5BB Kober, 101-14 Millardet et de Grasset, 161-49 Couderc and Fercal are examples of common female rootstocks. Among them, five common rootstocks in Spain were shown to be fertile in semi-natural conditions (5BB Kober: 24.4 seeds per bunch (SPB), 161-49 Couderc: 76.6 SPB, 19-62: 17.9 SPB, 41B MGT: 18.7 SPB and 1202C: 28.8 SPB [25]). Those varieties can therefore reproduce in a vegetative or a sexual way. Production of seeds depends on pollen availability, which may have many origins: (I) the rootstock itself, (II) an adjacent naturalised male population or (III) the European cultivars. The rootstock may self-pollinate depending on its hermaphrodism rate (expected to be low, Reisch and This, personal communication). An adjacent anthropogenic escaped population constituted by male rootstocks may be the paternal parent. Its presence would depend on composition and arrangement of rootstocks in vineyards. European cultivars could also pollinate anthropogenic escaped populations. The low genetic barriers are exploited by breeders to produce interspecific varieties. Sexual reproduction would probably lead to a huge diversity of genotypes in such populations, as *Vitis* cultivars are known for their high level of heterozygosity [26].

Natural escaped populations benefit from the dispersal strategy of the species and the existing connectivity of the landscape. Those populations are the direct consequence of an invasive process followed by escaped species. The settlement site is distant from vineyards and requires a long distance colonisation event. On the local scale, the presence of natural escaped populations directly depends on geomorphological and landscape structure parameters.

These populations of rootstocks can be located in convergence zones such as alluvial regions, allowing several different rootstock varieties to meet. The huge available diversity of rootstocks is revealed once the plant escapes and crosses with other naturalised individuals. With 19 different genotypes out of 23 individuals, our results may suggest that this phenomenon is already under process. Consequently, these populations are likely to acquire a large genetic diversity in a short term, depending on their location and potential contacts with other naturalised individuals.

### Genetics

Our results show that naturalised rootstocks and wild grapevine individuals clearly belong to two distinct genetic pools, confirming previous results [27]. Moreover, no hybridised or introgressed individual could be detected in our sampling. Consequently, we assume that our diversity measurements are not likely to be biased by including hybrids in one or the other pool, which would artificially increase the genetic diversity indices.

The ecological niche of European wild grapevines is large and complex. This may explain the high genetic diversity indices shown by our results and by other European studies [26], [28]. Interestingly, rootstocks show even higher diversity indices even though they are nearly restricted to alluvial zones. This high observed diversity clearly outlines the wide geographic origins of the naturalised rootstocks compared to European wild grapevines [29]–[31]. Indeed, rootstocks are obtained by selection and crosses of *Vitis* species of American, Asian and European origin, thus mixing a large pool of genes.

Naturalised rootstock populations show additional interesting features, they possess many traits of vine growing interests. Rootstock varieties were developed to fit a given environment, e.g. *V. berlandieri* is used in crossings for its tolerance to calcareous soils. Additionally, resistances to cultural pests and diseases such as phylloxera, downy mildew or powdery mildew may confer to naturalised rootstocks a strong competitive advantage compared to the native taxa, if a selection pressure is present.

Several studies outlined the wide genetic diversity of non-native species in the United-States (reviewed by Ellstrand et al. (2000) [32]). Non-native species generally arise from multiple introduction events. They provide genotypes and alleles from disparate sources. If introduced populations spread and coalesce, there is a “great opportunity for hybridisation among these independent lineages”. Indeed, hybrid-derived populations are found to have more genetic variation than parent species. Such hybridisation events may explain the origin of new invading species.

### Conclusion

The four different objectives of the paper were adressed. Naturalised rootstocks are present in the ecological niche of *V. vinifera* ssp. *silvestris*. Especially in alluvial zone, they compete with the native taxa and are able to compromise its survival. As viticulture represents constant sources of new alien populations, this phenomenon concerns European vineyards near to alluvial ecosystems. Current naturalised populations are well established in these functional webs, and they may not have yet colonised the entire river network, but have the potential to do so. Modelling studies should be conducted to better define the potentially invaded sites.

The accumulation of rootstocks in natural escaped populations tends to create hybrid swarms of rootstocks. These progenies have a huge diversity and benefit from exchanges of several genes of viticultural interest. Thus new introduced genes in viticulture environment may spread in the naturalised rootstock network, leading to a rapid loss of control of escaped genes. These latter populations may represent a clear danger and should remain under control via appropriate management measures:

- Properly define the identity of wild grapevines in order to avoid misidentifications or refer to *Vitis* specialists for a clear identification of individuals.
- Eliminate naturalised populations by considering the escape process: (I) vineyards borders, (II) hedges, (III) alluvial zones.

Many important questions are proposed to stimulate researches about this complex situation. Will the naturalised rootstocks pool widen its ecological niche, especially in the context of its ability to exchange genes of interest? What would be the consequences of such exchange?

At the moment, no crosses between wild grapevines and naturalised individuals have been found. As sympatry between both taxa is a reality, questions about genetic barriers are of concern. Do they rely on sexual incompatibility, or are ecological causes (such as phenology mismatches) involved?

Naturalised rootstocks must be controlled. Their presence in the landscape is a consequence of human activity and they should be treated as an invading species before representing a real threat.

## Materials and Methods

### Field data collection

The study area includes the Rhone and the Durance Valleys, extending from the Alps (Valais, Switzerland) to the Mediterranean Sea (Bouches-du-Rhône, France). Only “natural” populations distant from vineyards were considered. A preliminary study based on a good knowledge of the general ecology of wild grapevines, known locations, vegetation surveys and maps [33]–[36] targeted areas with high potential.

Each sample location was recorded by GPS. Pictures of the site and of some individuals were taken. Ecological data such as slope (%, quantitative), exposition (NSEW or flat, binomial categories), area covered by the population (m^2^, quantitative), vegetation type (forest, forest edge or clearing, binomial categories), vertical structure of the grapevine on tree, shrub or grass strata (semi-quantitative), distance to the water level (m, quantitative), type of stream (river, rivulet, canal, river branch, lake or absence of water nearby, binomial categories) were collected for each site.

In the field, four different habitats could be easily differentiated and designated as hedges, riparian forests, streamside hedges and screes, according to Delarze et al. [37]. The 20 ecological variables were investigated with an ordination (PCoA calculated with a Gower similarity index, Figure 2). The habitats were not included as variables in the analysis, but were used to label the ordination, which confirmed their distinct identities.

### Plant Material

Morphological identification of European grapevines from Asian or American rootstocks was based on phenotypic traits [2], [38]. For instance, the American rootstocks have broader than larger trilobate leaves. Each of the three lobes ends in a narrow apex, and the underside is glabrous. The sinus is widely open at the insertion of the petiole. The stems and petioles are reddish. The plant is dioecious. In autumn, it forms short bunches (10 cm), of round black berries.

The floral morphology, leaf characteristics and variation associated with *V. vinifera* ssp. *silvestris* [2] leads to many errors in vegetation surveys. General trends for leaf morphology can still be observed for European wild grapevines, despite the existing variability. Leaves are small, five-sided, and hairy on the lower surface. Pips are small and round, with an apiculate apex [39]. Chalazal ornamentation lies in the centre of the dorsal face, and the carina is deeply shaped on the ventral face. Naturalised European cultivars return to wild phenotypes and are thus difficult to distinguish; however the remaining individuals still have hermaphroditic flowers and cultivar shaped pips (pers. obs.). We do not consider these in the current study.

Fresh leaves were sampled and directly stored in silica-gel. A preliminary identification of grapevines was performed in the field and confirmed by our SSR analysis. The sampling is exhaustive for populations of *V. vinifera* ssp. *silvestris* and one to four individuals were collected in the naturalised rootstock populations. No European cultivars were detected in our dataset. Fifty-five populations containing either wild grapevines or rootstocks were selected for the current study, representing 124 individuals. A total of 20 common cultivated rootstocks were included in the analysis. This reference dataset was provided either by our own SSR analysis (Asia1–*V. amurensis*, US1–SO4 cl 5, US2– Rupestris du lot cl 110, US3-Riparia gloire cl 1, US4–Richter 110 cl 7, US5–*V. aestivalis*) or by consulting the Swiss Vitis Microsatellite Database [40] (Grézot cl 1, Fercal cl 242, Couderc 3309 cl FVA3, Dufour cl 11F, 3006-1, Couderc 161-49 cl 176, Gravesac, Mgt. 41B cl 153, Kober 5BB cl 114, Mgt. 101-14, Kober 125 AA cl 136, Mgt. 420-A cl 10, Teleki cl 8b, and Teleki 5C cl 236). The SVMD dataset was not included in figure 3, as only six SSR markers were available online.

### DNA amplification and GeneScan

DNA was extracted from dried leaves, following the CTAB extraction protocol from Rogers and Bendich (1985) [41]. Polyvinylpyrrolidon (PVP) was added to remove Polyphenols and 0.75 M Ammonium acetate was used to increase DNA purification.

DNA amplifications were done in a 15 μl reaction volume, with 1 μl of DNA, 1.5 mM MgCl_2_, 0.4 U *Taq* polymerase (promega), 1×PCR buffer, 200 μM of dNTP, 0.5 mg/ml Beef Serum Albumin and 20 pM primers.

Our primer set was constituted with six SSR primers, chosen as a core-set by the “International Grape Genetics Community” [42] (VVS2, VVMD5 VVMD7, VVMD27, ssrVrZAG62 and ssrVrZAG79) and was completed with four additional markers (VVMD6, VVMD17, VVMD21 and VVMD25 [27], [29], [43], [44]).

One out of each primer pair was labelled with either 6-FAM, PET, VIC or NED (Applied Biosystems). Amplification products were diluted ten fold before running GeneScan. Genotyping was performed in a four-colour multiplex using an ABI3100 sequencer. The raw data set was interpreted with GeneScan 3.7 and Genotyper 3.7 [45], [46].

### Statistical analysis

The SSR dataset was used to separate the European wild grapevines from the rootstocks. For this purpose, three methods were confronted: an ordination applied on a Jaccard similarity index (calculated on transformed SSR dataset-presence/absence format of each allele), a non-hierarchical clustering method (K-means, applied on the same transformed SSR dataset, with two groups assumed and 1000 iterations) and finally a bayesian-clustering method (Structure 2.0 [47], assumptions: two groups, admixture model with standard settings, 200000 Burn-in period and 1000000 Reps). Those three approaches revealed that naturalised rootstocks and *V. vinifera* ssp. *silvestris* were clustered in two distinct genetic pools (Figure 3). These latter were then compared to the four habitat categories highlighted by the field prospecting and the analysis of the ecological dataset via a contingency table (Table 1).

Finally, general statistics were calculated (on the non-transformed SSR dataset) for both grapevine pools in order to better understand the genetic features of the naturalised rootstocks (Table 2). Our sampling method restricted the use of population genetic statistics, as only one to four individuals were sampled in populations of naturalised rootstocks. We therefore chose to avoid the use of population genetic statistics (such as F-statistics) and focused on general statistics performed on each pool separately: the naturalised rootstocks group versus *V. vinifera* ssp. *silvestris* group.

We used the following statistical programmes: FSTAT [48], Genetix [49] and MSA [50]. Measured indices were: observed heterozygosity (H.obs), Shannon's index of diversity [50], Number of alleles per locus (Rs–Allelic Richness independent of sample size [51]), and variation of repeats in the SSR motif (VarRepSSR-independent of sample size [50]). Statistical significance was based on the mean overall loci and tested with the non-parametric test of Wilcoxon. R (CRAN) [52] was used for data handling and tests performing.

## Supporting Information

Map S1 Distribution of the studied populations. Distribution of natural and anthropogenic escaped populations of rootstocks, within the studied area. (This KML file can be viewed with the Google Earth mapping system.)
(0.00 MB ZIP)
